# A Leaf‐Patchable Reflectance Meter for In Situ Continuous Monitoring of Chlorophyll Content

**DOI:** 10.1002/advs.202305552

**Published:** 2023-10-05

**Authors:** Kaiyi Zhang, Wenlong Li, Haicheng Li, Yifei Luo, Zheng Li, Xiaoshi Wang, Xiaodong Chen

**Affiliations:** ^1^ Innovative Center for Flexible Devices (iFLEX) School of Materials Science and Engineering Nanyang Technological University 50 Nanyang Avenue Singapore 639798 Republic of Singapore; ^2^ Institute of Materials Research and Engineering (IMRE) Agency for Science, Technology and Research (A*STAR) 2 Fusionopolis Way, Innovis #08‐03 Singapore 138634 Republic of Singapore

**Keywords:** chlorophyll content monitoring, plant wearable sensors, reflectance‐based measurements, smart agriculture

## Abstract

Plant wearable sensors facilitate the real‐time monitoring of plant physiological status. In situ monitoring of the plant chlorophyll content over days can provide valuable information on the photosynthetic capacity, nitrogen content, and general plant health. However, it cannot be achieved by current chlorophyll measuring methods. Here, a miniaturized and plant‐wearable chlorophyll meter for rapid, non‐destructive, in situ, and long‐term chlorophyll monitoring is developed. The reflectance‐based chlorophyll sensor with 1.5 mm thickness and 0.2 g weight (1000 times lighter than the commercial chlorophyll meter), includes a light emitting diode (LED) and two symmetric photodetectors (PDs) on a flexible substrate, and is patched onto the leaf upper epidermis with a conformal light guiding layer. A chlorophyll content index (CCI) calculated based on the sensor shows a better linear relationship with the leaf chlorophyll content (r^2^ > 0.9) than the traditional chlorophyll meter. This meter can wirelessly communicate with a smartphone to monitor the leaf chlorophyll change under various stresses and indicate the unhealthy status of plants for long‐term application of plants under various stresses earlier than chlorophyll meter and naked‐eye observation. This wearable chlorophyll sensing patch is promising in smart and precision agriculture.

## Graphical abstract

A miniaturized and plant‐patchable chlorophyll meter is developed for in situ and long‐term monitoring of plant health. The measurements and data collection can be easily realized by communicating with a smartphone wirelessly. It can indicate the abnormal physiological status of plants in advance of traditional chlorophyll meter and naked‐eye observation, making it a promising tool for smart agriculture.

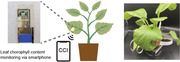

## 1 Introduction

Emerging plant‐wearable sensors allow for timely communication with plants to understand their physiological status, including temperature,^[^ ^1^ ^]^ water status,^[^ ^2^ ^]^ volatile emissions,^[^ ^3^ ^]^ and plant growth.^[^ ^4^ ^]^ They play a crucial role in providing data‐driven insights to optimize the growing conditions and prevent potential problems,^[^ ^5^ ^]^ ultimately resulting in higher yields and improved sustainability.^[^ ^6^ ^]^ The development of these wearables has the potential to revolutionize agriculture and horticulture.^[^ ^7^ ^]^ However, there are remaining challenges in monitoring the chlorophyll content in plants, which is also an important biomarker for plant health.

Chlorophylls, including chlorophyll *a* and chlorophyll *b*, are crucial pigments participating in photosynthesis. In the light‐dependent reaction of photosynthesis, chlorophyll absorbs light energy and converts it into chemical energy in the form of adenosine triphosphate and nicotinamide adenine dinucleotide phosphate which are used to assemble carbohydrate molecules in subsequent steps.^[^ ^8^ ^]^ The chlorophyll content is directly related to photosynthetic potential and primary production.^[^ ^9^ ^]^ Moreover, chlorophyll content is proportional to thylakoid nitrogen^[^ ^10^ ^]^ and is influenced by plant stress and senescence.^[^ ^11^ ^]^ Compared with current plant‐wearable sensors focusing on leaf humidity, temperature, and volatile organic compounds,^[^ ^4^, ^12^ ^]^ leaf chlorophyll content can provide more direct and insightful information on chloroplast development, photosynthetic capacity, leaf nitrogen content, or general plant health status.^[^ ^13^ ^]^

Traditional methods to measure the concentration of chlorophyll in leaves include wet chemical extraction and a portable clamp device called Soil Plant Analysis Development (SPAD) meter. In wet chemical extraction, chlorophyll is extracted from ground leaf tissues using organic solvents (e.g., acetone^[^ ^14^ ^]^ and N, N’‐dimethylformamide^[^ ^15^ ^]^). The chlorophyll concentrations are then calculated by measuring the absorption of the solution in a spectrophotometric way. This method can accurately measure the concentrations of chlorophyll *a* and *b*, but is complicated (grinding and filtering), time‐consuming (24–72 h), and destructive to leaves. Moreover, this method is limited to lab measurement and impractical for large amounts of samples in the field. Alternatively, a handheld chlorophyll meter (SPAD‐502)^[^ ^16^ ^]^ can perform non‐destructive quick measurements of leaf chlorophyll content. The SPAD meter clips on a leaf and measure light transmittance through the leaf using one photodetector (PD) and two light emitting diodes (LEDs) with wavelength at ≈650 nm and 940 nm. The chlorophyll content of the clipped leaf area is reflected as SPAD values.^[^ ^17^ ^]^ However, the intrinsic linearity between SPAD value and chlorophyll is poor,^[^ ^17^, ^18^ ^]^ increasing the difficulties in chlorophyll content evaluation. The manual clamping of the SPAD probe on the leaf for every measurement further causes significant operational bias.^[^ ^19^ ^]^ Recently a wearable sensor for chlorophyll monitoring has been developed based on fluorescence, but not all part of the sensor is wearable and the spectrometer is still involved in the measurement.^[^ ^20^ ^]^ In short, there is no method for non‐invasive, in situ, long‐term, and quick plant chlorophyll measurement, which limits in‐field plant health monitoring.

As a photosynthetic pigment, chlorophyll shows absorption maxima at wavelengths corresponding to blue light (450–475 nm) and red (650–675 nm) light, while reflects most of green light (500–550 nm). The measurement of reflected green light can provide an alternative way for evaluating chlorophyll content in the leaf. Several chlorophyll indices (e.g., Normalized Difference Vegetation Index (NDVI),^[^ ^21^ ^]^ Simple Ration of Reflectance,^[^ ^22^ ^]^ Chlorophyll Absorption Ration Index (CARI)^[^ ^23^ ^]^ have been developed from the leaf reflectance.^[^ ^24^ ^]^ However, most of these works focused on remote sensing and used precision instrument, such as spectrophotometer, to realize the measurement of chlorophyll indices.

**Figure 1:**
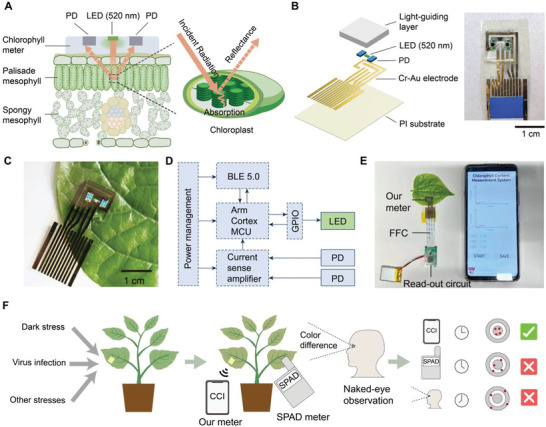
Overview of plant‐patchable chlorophyll meter based on reflective optics. A) Schematics of the working mechanism of the patchable chlorophyll meter. B) Explosive view and photograph of the patchable chlorophyll meter. C) Photograph of the wearable chlorophyll meter patched on the leaf. D) System block diagram of device operation. E) Wireless and portable platform based on smartphone for rapid and convenient measurements and data collection. FFC, flexible flat cable. F) The advantages of the patchable chlorophyll meter for early detection of plant stresses over naked‐eye observation and commercial SPAD meter.

In this work, we designed a miniatured, flexible, and wearable chlorophyll meter capable of in situ, long‐term plant monitoring, which was inspired by these reflectance‐based indices. It employs a monochromatic LED and a pair of symmetric PDs for incident radiation and measurement of the intensity of the reflected light. The chlorophyll content is calculated based on the relationship between leaf chlorophyll content and spectral reflectance (**Figure** **1**A,B). This meter is 1.5 mm thick and weighs 0.2 g, making it 1000 times lighter than the commercial chlorophyll meter. It can be patched onto the upper epidermis of the leaf tightly (Figure 1C) and realize long‐term monitoring with little negative impact on leaves and plants. The block diagram in Figure 1D summarizes the critical components of the meter and the read‐out circuit. Based on it, a smartphone‐controlled platform is developed for users to conduct measurements and collect data easily (Figure 1E). The power consumption of the system is 0.035 W. With our plant‐patchable chlorophyll meter, the leaf chlorophyll content can be measured more accurately and precisely (r^2^ > 0.9) than the SPAD meter. Moreover, during long‐term monitoring (over 2 weeks), chlorophyll losses due to abnormal physiological activities of plants can be detected earlier than the SPAD meter and naked‐eye observation of yellowing (Figure 1F).

## 2 Results and Discussion

The principle of the patchable chlorophyll sensor is based on the optical characteristics of leaves, but unlike the SPAD meter, leaf reflectance is used considering the wearable design of our chlorophyll meter (Figure S1, Supporting Information).^[^ ^25^ ^]^ The chlorophyll meter can be directly attached to the upper epidermis of a leaf, and a mono‐color LED with light wavelength at 520 nm is selected as most of the light interacts with the chlorophylls in chloroplasts. The intensity of the reflected light from the leaf is measured by the symmetric photodetectors (Figure 1A). The explosive structure of the chlorophyll meter is shown in Figure 1B, along with the real photos showing the front view of the meter. A conformal and transparent light‐guiding layer (LGL) is designed for efficient light‐receiving and stable contact with the leaf surface (Figure 1C).

The relationship between leaf chlorophyll content and spectral reflectance has long been investigated and it has been found that leaf reflectance is closely related to leaf chlorophyll level.^[^ ^26^ ^]^ For leaf with higher chlorophyll content, the leaf reflectance is lower in the visible spectrum.^[^ ^27^ ^]^ Various indices for leaf chlorophyll content assessment have been developed based on this relationship, among which the reciprocal reflectance is one of the most typical indices.^[^ ^9^, ^22^, ^28^ ^]^ Reciprocal reflectance in spectral ranges from 525 to 555 nm and from 695 to 725 nm is linearly proportional to the total chlorophyll content, despite the complicated optical structures of leaves.^[^ ^9a^ ^]^ Our chlorophyll meter takes advantage of this linear correlation. For the Si PIN photodiode employed in the meter, the short circuit current is linear with the light intensity and the range of linear operation is large, so here we use reciprocal photocurrent generated by the PDs from the reflected light 1/I_R_ in place of the reciprocal reflectance 1/R. As commercial LEDs are employed in this research, by giving a fixed voltage and current, the light intensity emitted from the LED (E_LED_) remains the same, and I_R_ is linear with the intensity of the reflected light E_R_ for photodiodes. In this way, 1/I_R_ is theoretically proportional to 1/R:

(1) $$\frac{1}{I_{R}}\propto \frac{1}{E_{R}}\propto \frac{E_{LED}}{E_{R}}\propto \frac{1}{R}$$

**Figure 2:**
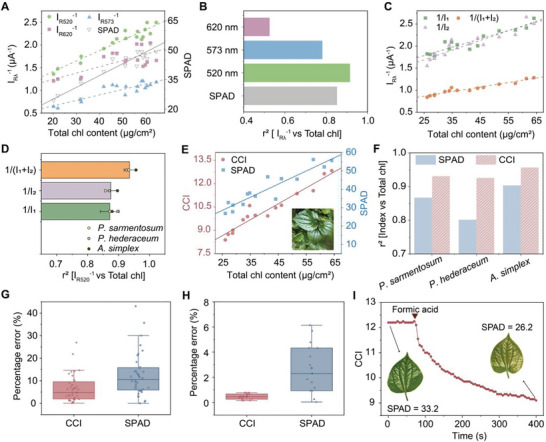
Evaluation on linear correlation between leaf total chlorophyll contents and leaf reflectance measured with different methods. A) Linear relationships of leaf total chlorophyll contents with SPAD value and reciprocal photocurrent using 520, 573, and 620 nm LEDs. B) The coefficient of determinations (*r* ^2^) of linear regressions of leaf total chlorophyll contents with SPAD value and reciprocal photocurrent using 520, 573, and 620 nm LEDs. C) Linear relationships of leaf total chlorophyll contents with reciprocal photocurrent using single PD and symmetric PDs on *Piper sarmentosum*. D) The coefficient of determination (*r* ^2^) of linear regressions of leaf total chlorophyll contents with reciprocal photocurrent using single PD and symmetric PDs on three species of plants. E) Linear relationships of leaf total chlorophyll contents with SPAD value and CCI value on *Piper sarmentosum*. F) The coefficient of determination (*r* ^2^) of linear regressions of leaf total chlorophyll contents with SPAD value and CCI value on three species of plants. The total chlorophyll contents were measured with wet chemical extraction method. G) Box plot of percentage errors from linear regression (median, 25th and 75th percentiles, minimum and maximum; *n* = 36 from three plants). H) Box plot of percentage errors of same‐point measuring (median, 25th and 75th percentiles, minimum and maximum; *n* = 15). I) Continuous monitoring on chlorophyll content of an acid‐treated leaf. Inset displays the leaf before acid treating and after acid treating.

Based on previously developed reflectance‐based indices for chlorophyll estimation, spectral band between 500 and 700 nm is most frequently used. Therefore, the spectral reflectance of 520 nm, 573 nm, and 620 nm were investigated on 17 leaves from *Piper sarmentosum*. Linear regression was used and the coefficient of determination (r^2^) was studied to evaluate the relationship between the reciprocal photocurrent and total chlorophyll content, as shown in **Figure** **2**A,B. The reciprocal photocurrent at 620 nm was essentially nonlinear with total chlorophyll content as r^2^ is ≈0.52, while reciprocal photocurrent at 520 nm (I_R520_^−1^) was correlated with total chlorophyll content most closely, with r^2^ over 0.9. The weak correlation between the reflectance at 620 nm and total chlorophyll contents may be due to the high absorption of chlorophylls. The leaf reflectance mainly includes specular reflectance and diffuse reflectance.^[^ ^29^ ^]^ The specular reflectance arises from the air‐cuticle interface, which is influenced by leaf surface features, while the diffuse reflectance emanates primarily from the leaf interior structures,^[^ ^30^ ^]^ which is dominated by the optical characteristics of the leaf pigments in the visible spectrum range. Most of red light as the incident radiation is absorbed by the chlorophylls so little diffuse reflectance can be measured, thus the specular reflectance cannot be neglected and weakens the correlation between the reflectance and total chlorophyll contents. Compared with the SPAD value, the linear relationship between total chlorophyll content and reciprocal photocurrent at 520 nm (I_R520_^−1^) was even stronger. The linear relationship can be further improved by employing symmetric PDs (Figure 2C,D). Therefore, for our meter, a chlorophyll content index is calculated as:

(2) $$\mathrm{CCI}=\frac{10}{I_{1}+I_{2}}\left(\mu A^{-1}\right)$$

in which I_1_ and I_2_ are photocurrents of two PDs. In measuring, the short circuit current of PDs is measured first under ambient light with the LED off as I_off_, and then with the LED on as I_on_. I_1_ and I_2_ are calculated as I_on_ minus I_off_, which helps to minimize the influence of ambient light. The measured CCI value is quite stable under different light environments with a percent error smaller than 0.04% (Movie S1, Supporting Information), which is also evidence proving the tiny variations of our chlorophyll meter when measuring the same point of a leaf. The solid linear correlation between CCI values and leaf total chlorophyll contents has been verified on three species of plants with different leaf surface morphologies (Figure 2E–G; Figure S2, Supporting Information), including *Piper sarmentosum* (*P. sarmentosum*), *Philodendron hederaceum* (*P. hederaceum*), and *Aglaonema simplex* (*A. simplex*). When measuring the same point of a leaf, the percentage error of our chlorophyll meter is ignorable (0.47%, Figure 2H), while the error of SPAD meter is 2.3% due to the manual operation. Therefore, we can conclude that our meter is able to measure the leaf chlorophyll content accurately with smaller errors. This sensor successfully realized continuous monitoring of leaf chlorophyll content on a leaf treated with 40% (v/v) aqueous formic acid (Figure 2I). Such continuous monitoring shows another advantage over SPAD meter.

**Figure 3:**
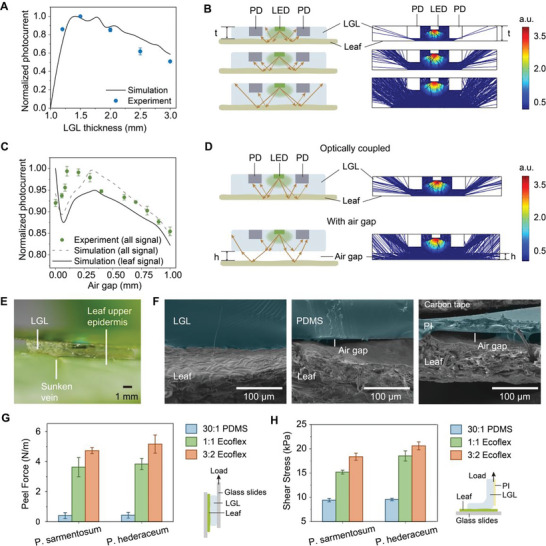
Characterization of light‐guiding layer. A) Normalized photocurrent of PDs generated from leaf‐reflected light with the thickness of LGL from 1.0 to 3.0 mm; *n* = 3 for experimental results. B) Illustrations and simulations of optical light paths with LGLs of different thicknesses. Left, schematic showing light reflections (orange line) at different LGL thickness. t, LGL thickness. Right, simulated result showing light paths. The color legend indicates the ray intensity. C) Normalized photocurrent of PDs generated from leaf‐reflected light with the air gap between LGL and leaf upper epidermis from 0 to 1 mm; The solid dot indicates mean and error bar indicates s.d., *n* = 3. D) Illustrations and simulations of optical light paths when the LGL and leaf upper epidermis are optically coupled and when the air gap exists. Left, schematic showing light reflections (orange line) at different air gap thicknesses. h, air gap thickness. Right, simulated result showing light paths. The color legend indicates the ray intensity. E) Photograph of the conformal patching of our chlorophyll sensor on the leaf. F) Cross‐sectional SEM images of LGL, PDMS and PI attached to the upper surface of Monstera deliciosa leaves. G) Histogram showing peel force of LGL made of three kinds of soft polymeric materials on two kinds of leaves. The bar indicates mean and error bar indicates s.d., *n* = 3. Inset displays the experimental setup of the peel test. H) Histogram showing shear stress of LGL made of three kinds of soft polymeric materials on two kinds of leaves. The bar indicates mean and error bar indicates s.d., *n* = 3. Inset displays the experimental setup of the shear test.

The LGL which encapsulates the components is a conformal and transparent cover, aims at providing stable and efficient light propagation. The surfaces of most leaves have irregular textures and are uneven. Those rough textures will influence the light propagation and bring about deviation in optical‐based measurement. The design of LGL can improve the intensity and accuracy of received signals from two factors: controllable distance between optoelectronic components (PD and LED) and the leaf surface, and optically coupled meter‐leaf interface. Here, the LGL is made of Ecoflex Gel due to its high transparency (Figure S3, Supporting Information) and good adhesive properties, which will be further explained below. First, to investigate how the thickness of the Ecoflex LGL influences the photocurrent signals, simulations were conducted through COMSOL and 15 devices with five different thicknesses of LGL were tested. The experimental result was generally in accordance with the simulation (**Figure** **3**A). With the increasing thickness, the photocurrent signals first increased and then decreased. This is because the thickness of the LGL changes the light path, thus changing the intensity and the falling point of reflected light, as illustrated in Figure 3B. When the thickness is small, most reflected rays fall on the part between LED and PD, so little reflected light is detected by PDs, even though the intensity of the light is relatively higher. Conversely, with a thick LGL, the light intensity is reduced, and meanwhile more reflected rays fall outside PDs. When the thickness is ≈1.5 mm, with the coordination between the intensity and the number of reflected rays falling on PDs, the total intensity of reflected light that the PDs received was the strongest.

Due to the irregular textures on the leaf surface,^[^ ^31^ ^]^ the air gap between the meter and the upper leaf surface is a common phenomenon. With the air gap, part of the light maybe received by PDs without going through the leaf, which will influence the intensity and accuracy of signals. Similarly, the relationship between the photocurrent and the air gap was investigated by simulation and experiments. The results are shown in Figure 3C. Air gaps with 12 different heights were tested in experiments, and the same conclusion can be drawn from simulation and experiments: the photocurrent first increased and then decreased with the increasing height of the air gap. The signal reached a maximum when there was a small air gap, however, part of the signal mainly came from the light guided only in the LGL (LGL signal), rather than the light that interacted with the leaf and reflected (leaf signal) as illustrated in Figure 3D. Such LGL signal overshadowed the real one and caused the inaccuracy of the output signal, even though the total intensity of the signal was higher.^[^ ^32^ ^]^ The leaf signal was calculated in the simulation. From the result it can be concluded that when the LGL is optically coupled to the leaf surface, the signals received by PDs are the most accurate and strongest. As the simulation suggests, the height of the air gap affected the accuracy of received signals, and in order to acquire the most accurate signals, it is important to eliminate the air gap. The close and conformal contact between the LGL and the leaf surface is observed from macro perspective in Figure 3E. To better prove the absence of the air gap, the cross‐section of the leaf‐device interface was studied through optical microscopy and scanning electron microscopy (SEM). The seamless contact between the Ecoflex LGL and the leaf surface of *Nicotiana benthamiana* and *Brassica chinensis* var. *parachinensis* is revealed (Figure S4A, Supporting Information). The SEM images show the conformal contact of LGL with leaf having surface textures, and with curved leaf surface as well (Figure 3F; Figure S4B, Supporting Information), which further proved a high degree of conformability of the LGL. Such conformable LGL attached to the leaf surface is capable to eliminate the air gap and improve the accuracy of signals acquired from plants. While for PDMS or PI substrates which are commonly used in plant‐wearable sensors,^[^ ^1^, ^2^, ^33^ ^]^ obvious air gaps are observed even with the help of adhesives (Figure 3F).

Apart from providing a conformal interface for efficient light receiving, LGL also performs as an adhesive layer to allow the meter to attach to the leaf tightly, especially in long‐term monitoring. To study the adhesive properties of LGL, we tested the sheer stress and peel force of LGLs made of three different transparent polymetric materials on leaves from 2 plant species.^[^ ^34^ ^]^ Representative peel force‐displacement and shear stress‐displacement curves are shown in Figure S5 (Supporting Information). As summarized in Figure 3H,I, PDMS adheres very weakly on both kinds of leaves, and Ecoflex gel with 3A:2B weight ratio shows higher shear stress and peel force than Ecoflex gel with 1A:1B weight ratio before being detached from leaves. Ecoflex gel (3A:2B weight ratio) can adhere tightly to leaves and allow the close contact between the meter and the leaf surface during long‐term monitoring. The Ecoflex gel has better adhesive properties over PDMS,^[^ ^35^ ^]^ meanwhile the 3:2 ratio by weight of part A: part B allows for a half‐cured status of the LGL when being adhered to the leaf upper epidermis, which can further seal the remaining air gap.^[^ ^36^ ^]^ For higher part A: part B ratio (2A:1B weight ratio), the fully cured status is difficult to be reached.

To demonstrate the superiority of the wearable chlorophyll meter over naked‐eye observation and the commercial SPAD meter, we monitored the chlorophyll change in living plants under different stresses. SPAD value is measured by the commercial SPAD meter. CCI value is measured with our patchable meter. The change of visual color is quantified by color difference ∆E_00_ in CIELAB color space which is calculated with the CIEDE2000 color difference formula.^[^ ^37^ ^]^ A just noticeable difference (JND) value is ∆E_00_ ≈2.3.^[^ ^38^ ^]^ Our meter is attached onto a leaf for 10 days and then detached from the leaf without causing any hurts. The meter‐attached point shows little difference from other parts of the leaf (Figure S6, Supporting Information), which shows that our meter is biocompatible to leaves without affecting their normal physiological activities.

**Figure 4:**
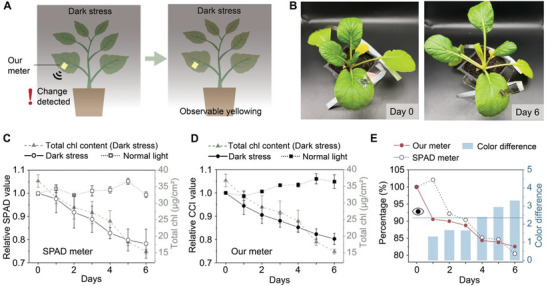
Non‐invasive, in situ, and long‐term monitoring of dark‐stress‐induced chlorophyll change by the patchable chlorophyll meter. A) Illustration of the monitoring for the dark stressed plant. B) Photographs of Brassica chinensis var. parachinensis under dark stress for 0 and 6 days. C) Relative SPAD values measured by commercial SPAD meter of plants in dark environment (<1 lux) and normal light environment (≈1600 lux) in 6 days, and total chlorophyll contents of dark‐stressed plants in 6 days. The dot indicates mean and error bar indicates s.d., *n* = 6. D) Relative CCI values measured by the patchable chlorophyll meter of plants in dark environment (<1 lux) and normal light environment (≈1600 lux) in 6 days, and total chlorophyll contents of dark‐stressed plants in 6 days. The dot indicates mean and error bar indicates s.d., *n* = 6. E) The trend of CCI values, SPAD values and color difference in CIE LAB space of the dark‐stressed plant in 6 days. The dark blue dotted line with an eye above refers to JND.

First, the change in chlorophyll content of plants under dark stress was monitored. Light is a crucial factor participating in photosynthesis and the synthesis of chlorophyll as well, so theoretically the chlorophyll content will decrease with dark stress.^[^ ^39^ ^]^ In the experiment, intact *Brassica chinensis* var. *parachinensis* plants were placed in a dark environment (light intensity < 1 lux) for six days (**Figure** **4**A). The CCI and SPAD values were measured daily through a smartphone and SPAD meter, respectively. We took photos of the the plant under the same light with the same photographic parameters (Figure 4B; Figure S7, Supporting Information). As shown in Figure 4C,D, the general tendencies are similar: both values declined over time, corresponding to chlorophyll content decline due to light deficiency. However, in the first day of dark stress, the change of relative SPAD value is quite small (≈0.02), while the decline of relative CCI value is about 0.06. The low sensitivity of SPAD may be due to the nonlinear relationship with the total chlorophyll content. When the chlorophyll content is high, the change of SPAD value will be small.^[^ ^13^, ^17^, ^18^, ^40^ ^]^ Moreover, our meter showed a smaller variation during monitoring. This is because our meter is miniatured, lightweight, and patchable to the leaf surface, which keeps the device‐plant interface intact and unchanged during monitoring, whereas we have to use the SPAD meter to clip the leaf every time for measurement, causing significant operational errors. To prove the advantage of our meter over naked‐eye observation, we got the CIE LAB values of the leaf part near the meter‐attached point and calculated the color difference ΔE_00_ with the first day. Figure 4E shows ΔE_00_ is less than 2.3 for both groups in the first 3 days, which means the color difference is difficult to be observed, while the CCI values have already shown a decline of ≈12%. It proves that our chlorophyll meter can detect the unhealthy status of plants before noticeable signs of observable yellowing.

**Figure 5:**
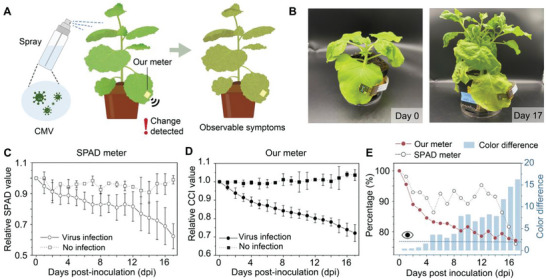
Non‐invasive, in situ, and long‐term monitoring of virus‐infection‐induced chlorophyll change by the patchable chlorophyll meter. A) Illustration of the monitoring for the virus‐infected plant. B) Photographs of CMV‐infected Nicotiana benthamiana plants at 0 and 17 dpi. C) Relative SPAD values measured by commercial SPAD meter of CMV‐infected plants in 17 days. The dot indicates mean and error bar indicates s.d., *n* = 6. D) Relative CCI values measured by the patchable chlorophyll meter of CMV‐infected plants in 17 days. The dot indicates mean and the error bar indicates s.d., *n* = 6. E) The trend of CCI values, SPAD values and color difference in CIE LAB space of the virus‐infected plant in 17 days. The dark blue dotted line with an eye above refers to JND.

We also monitored *Nicotiana benthamiana* infected by cucumber mosaic virus (CMV). After being infected by the virus, the primary cellular metabolic functions will be altered to support the viral genome replication, in which photosynthesis is included,^[^ ^41^ ^]^ causing chlorophyll loss in infected plants. The monitoring was conducted for 17 days (**Figure** **5**A), and the visual leaf color (Figure 5B), SPAD value (Figure 5C), and CCI value (Figure 5D) were recorded with the similar methods of dark‐stressed plants. The decline of relative SPAD value is small with large variation, and the stable decline can only be observed at 10 dpi (days post inoculation). While for our chlorophyll meter, the everyday decline is obvious and stable throughout 17 days. It proves that our meter can detect the unhealthy status earlier than the SPAD meter. Similarly, the color difference was calculated and compared with CCI and SPAD value (Figure 5E). At four dpi, the color difference is 1.59, whereas the CCI value has already dropped 15.5%, compared with a 9.0% decline in SPAD value. Moreover, from photos recorded (Figure S8, Supporting Information), the leaf at four dpi showed little infection symptoms including leaf distortion and yellowish patches.

Therefore, we can conclude that our meter is able to detect the abnormal physiological status before visible symptoms can be observed. It allows for timely intervention and prevents the condition from deteriorating even further. Besides, for green leafy vegetables, chlorophyll degrades during storage after harvesting, so chlorophyll content can be used to evaluate the quality and freshness of these vegetables.^[^ ^42^ ^]^ The wearable chlorophyll meter also showed a potential application in freshness detection of green leafy vegetables (Figure S9, Supporting Information). Our plant‐patchable showed advantages over the commercial SPAD meter in size and application. The more detailed comparison is summarized in Table S1 (Supporting Information). This meter has potential application in precise and smart agriculture and horticulture.

## 3 Conclusion

We explored the non‐destructive measurement of leaf chlorophyll content based on leaf reflectance. A miniatured, flexible, and plant‐patchable chlorophyll meter has been developed based on reflective optics, and a smartphone‐based wireless platform has been established for rapid measurement and convenient data collection. Our wearable chlorophyll meter is capable of measuring the chlorophyll content in situ and monitoring the plant health status in a long term. Compared with the commercial SPAD meter, the size and weight of our device are significantly reduced, and the conformable and adhesive LGL of our meter provides a stable leaf‐device interface and shows more accurate and sensitive detection in in situ and long‐term plant monitoring.

## 4 Experimental Section

### Fabrication of Miniaturized and Patchable Chlorophyll Meter

First, the Cr‐Au electrode was deposited onto the transparent polyimide substrate by thermal evaporation. The thickness of chromium/gold is 10/100 nm. The thickness of the PI substrate was 35 µm. Second, one light‐emitting diode (WL‐SMCW SMT Mono‐color Chip LED Waterclear) and two photodiodes (HAMAMATSU S10993‐05T) were connected symmetrically to the gold electrode with liquid metal. Third, a silicon mold with a square hole (1 cm × 1 cm) was attached onto the PI substrate. 1A:1B and 3A:2B mixed (weight ratio) Ecoflex Gel and 30:1 PDMS were centrifuged at 8000 rpm for 3 min to remove the air bubbles. Then it was poured into the square hole of the silicon mold. After heating in oven at 60 °C (2 h for PDMS and 3 min for Ecoflex Gel), remove the silicon mold. In this way, a 1‐cm square colorless conformal cover encapsulated the liquid metal and LED and PD components. Then the device was attached to the upper leaf surface immediately. Another 1 h at room temperature was needed for the Ecoflex Gel to be fully cured.

### Plant Materials and Growth Conditions

For chlorophyll measurement, the leaves of *Piper sarmentosum*, *Aglaonema simplex*, and *Philodendron hederaceum* were freshly picked outdoors. Leaves with different chlorophyll contents were selected according to their color. For every species of plant, 15 leaves were selected and washed with deionized water to remove dust and were dried with tissues before measuring. For plant monitoring, intact plants of *Brassica chinensis* var. *parachinensis* and *Nicotiana benthamiana* were used. They were grown in an environment with 1600 lux light intensity, 60% relative humidity, 25 °C. Plants were grown for a month before experimental use. For vegetable freshness detection, fresh *Brassica rapa* subsp. *chinensis* was purchased from a local supermarket in Singapore. Leaves were washed with deionized water to remove dust and were dried with tissues before measuring. The leaves chosen for experimental use were all healthy and visually homogeneous in color.

### Measurement of Total Chlorophyll Concentration

The total chlorophyll content was measured with wet chemical extraction method. To be more detailed, after the reflectance was measured, leaves were cut into small pieces with length ≈1 mm, and pigments were extracted with dimethyl sulfoxide (DMSO). The absorbance spectra of the resulting solutions were analyzed by UV–vis spectrophotometer, and the total chlorophyll concentrations (C_a+b_) were calculated as:

(3) $$C_{a+b}=7.49A_{664.9}+20.34A_{648.2}$$

in which A_664.9_ and A_648.2_ refer to absorbance at 664.9 nm and 648.2 nm wavelengths respectively.^[^ ^43^ ^]^

### Design and Fabrication of Light Guiding Layer (LGL)

The optimization of the device took two parameters into account: the thickness of the Ecoflex LGL and the air gap between the surface of the Ecoflex LGL and the leaf upper epidermis. In experiments, the thickness of Ecoflex LGLs was controlled by silicon mold with 1.2 mm, 1.5 mm, 2.0 mm, 2.5 mm, and 3.0 mm thickness. Three devices were tested for every thickness. The height of the air gap between the Ecoflex LGL and the surface of testing samples was controlled by the height of PTFE pads between them. Three devices were tested for every height of air gap. Device simulations aimed at understanding the device physics better. Ray Optics Module in COMSOL Multiphysics 5.4 was used for ray tracing and intensity analysis. In simulation, the leaf was regarded as a 2D plane. The optical property of the surface was set as mixed diffuse‐specular reflection with 0.67 absorption coefficient.^[^ ^44^ ^]^

### Cross‐Sectional SEM of Leaf‐Device Interface

For scanning electron microscopy, the healthy leaves of *Monstera deliciosa* were freshly picked and then freeze dried for 24 h. The LGL was attached onto the freeze‐dried leaves before the SEM imaging.

### Measurement of Chlorophyll Content Index (CCI)

Attach the LGL onto the clean surface of the leaf for measurement. The meter was connected to a customized read‐out circuit board (1.5 cm × 2.5 cm) with a flexible flat cable (FFC). In long‐term monitoring this circuit board along with its battery was attached onto the flowerpot without adding additional weight onto leaves. The read‐out circuit was BLE integrated, so measuring and data collecting can be conducted through wireless communication via an application for Android mobile phones. After connecting the device with this app via Bluetooth, press the “Start” button. It would collect the dark signals of both PDs for 1 s. Then the green LED was turned on, and the app received the total currents of the two PDs for another 1 s. The measurement lasts for 2 s, and then a chlorophyll content index (CCI) will be shown on the mobile phone. Signals received within the 2 s can be saved to the phone. With the smartphone‐controlled platform, users can conduct measurements and collect data through a mobile phone easily. The percentage error of SPAD and CCI values were calculated as:

(4) $$Percentage\;error=\frac{\left|Chl_{index}-Chl\right|}{Chl}\times 100\%$$

where *Chl_index_* is the calculated chlorophyll content from SPAD and CCI with the linear regression formula, *Chl* is the chlorophyll content measured with wet chemical extraction method.

### Dark Stress and Virus Infection

Dark stress was performed by placing the intact choy sum plants in an air‐permeable box covered with shading cloth. The light intensity was lower than 1 lux for 7 days. Cucumber mosaic virus (CMV) was inoculated to intact Nicotiana benthamiana plants by spraying virus solution to the upper surface of the leaves which had been bruised by carborundum previously.^[^ ^45^ ^]^ These directly inoculated leaves were not selected to be used in monitoring experiment for CCI and SPAD measuring.

## Conflict of Interest

The authors declare no conflict of interest.

## Supporting information

Supporting Information

Supplemental Movie 1

## Data Availability

The data that support the findings of this study are available from the corresponding author upon reasonable request.

## References

[1] J. M. Nassar , S. M. Khan , D. R. Villalva , M. M. Nour , A. S. Almuslem , M. M. Hussain , npj Flexible Electron 2018, 2, 24.

[2] a) H. Im , S. Lee , M. Naqi , C. Lee , S. Kim , Electronics 2018, 7, 114;

[3] a) Z. Li , Y. Liu , O. Hossain , R. Paul , S. Yao , S. Wu , J. B. Ristaino , Y. Zhu , Q. Wei , Matter 2021, 4, 2553;

[4] a) W. Tang , T. Yan , F. Wang , J. Yang , J. Wu , J. Wang , T. Yue , Z. Li , Carbon 2019, 147, 295;

[5] J. M. Buriak , L. M. Liz‐Marzán , W. J. Parak , X. Chen , ACS Nano 2022, 16, 1681.

[6] G. Lee , Q. Wei , Y. Zhu , Adv. Funct. Mater. 2021, 31, 2106475.

[7] a) C. C. Qu , X. Y. Sun , W. X. Sun , L. X. Cao , X. Q. Wang , Z. Z. He , Small 2021, 17, 2104482; 10.1002/smll.202104482 34796649

[8] P. Gan , F. Liu , R. Li , S. Wang , J. Luo , Int. J. Mol. Sci. 2019, 20, 5046. 31614592 10.3390/ijms20205046 PMC6834309

[9] a) A. A. Gitelson , Y. Gritz † , M. N. Merzlyak , J. Plant Physiol. 2003, 160, 271; 12749084 10.1078/0176-1617-00887

[10] J. R. Evans , Oecologia 1989, 78, 9. 28311896 10.1007/BF00377192

[11] a) D. Jespersen , J. Zhang , B. Huang , Plant Sci 2016, 249, 1; 27297985 10.1016/j.plantsci.2016.04.016

[12] a) A. Afzal , S. W. Duiker , J. E. Watson , D. Luthe , Trans. ASABE 2017, 60, 1063;

[13] Q. Ling , W. Huang , P. Jarvis , Photosynth. Res. 2011, 107, 209. 21188527 10.1007/s11120-010-9606-0

[14] D. I. Arnon , Plant Physiol. 1949, 24, 1. 16654194 10.1104/pp.24.1.1 PMC437905

[15] R. Porra , W. Thompson , P. Kriedemann , Biochimica et Biophysica Acta (BBA)‐Bioenergetics 1989, 975, 384.

[16] K. Minolta , SPAD‐502 Plus, https://www.konicaminolta.eu/eu-en/hardware/measuring-instruments/colour-measurement/chlorophyll-meter/spad-502plus (accessed: June 2023).

[17] J. Uddling , J. Gelang‐Alfredsson , K. Piikki , H. Pleijel , Photosynth. Res. 2007, 91, 37. 17342446 10.1007/s11120-006-9077-5

[18] a) J. Markwell , J. C. Osterman , J. L. Mitchell , Photosynth. Res. 1995, 46, 467; 24301641 10.1007/BF00032301

[19] a) Y. Wang , D. Wang , P. Shi , K. Omasa , Plant Methods 2014, 10, 36; 25411579 10.1186/1746-4811-10-36 PMC4236477

[20] R. M. Galatus , R. Papara , L. Buzura , A. Roman , T. Ursu , presented at Biophotonics in Point‐of‐Care, 2020.

[21] a) J. W. Rouse Jr , R. H. Haas , J. Schell , D. Deering , 1973;

[22] a) E. W. Chappelle , M. S. Kim , J. E. McMurtrey, III , Remote Sens. Environ. 1992, 39, 239;

[23] M. S. Kim , C. Daughtry , E. Chappelle , J. McMurtrey , C. Walthall , presented at CNES, proceedings of 6th int. symp. on physical measurements and signatures in remote sensing, CNES, Paris France 1994.

[24] R. Sonobe , T. Sano , H. Horie , Biosyst. Eng. 2018, 175, 168.

[25] a) Y. Ling , T. An , L. W. Yap , B. Zhu , S. Gong , W. Cheng , Adv. Mater. 2020, 32, 1904664; 10.1002/adma.201904664 31721340

[26] a) B. Cui , Q. Zhao , W. Huang , X. Song , H. Ye , X. Zhou , Remote Sens 2019, 11, 974;

[27] a) D. Lamb , M. Steyn‐Ross , P. Schaare , M. Hanna , W. Silvester , A. Steyn‐Ross , int. j. remote sensing 2002, 23, 3619;

[28] a) G. A. Carter , A. K. Knapp , Am. J. Bot. 2001, 88, 677; 11302854

[29] C. R. Brodersen , T. C. Vogelmann , Am. J. Bot. 2007, 94, 1061. 21636475 10.3732/ajb.94.7.1061

[30] L. Grant , Remote Sens. Environ. 1987, 22, 309.

[31] J. T. Woolley , Plant Physiol. 1971, 47, 656. 16657679 10.1104/pp.47.5.656 PMC396745

[32] H. Lee , E. Kim , Y. Lee , H. Kim , J. Lee , M. Kim , H.‐J. Yoo , S. Yoo , Sci. Adv. 2018, 4, eaas9530. 30430132 10.1126/sciadv.aas9530 PMC6226280

[33] Y. Lu , K. Xu , L. Zhang , M. Deguchi , H. Shishido , T. Arie , R. Pan , A. Hayashi , L. Shen , S. Akita , ACS Nano 2020, 14, 10966. 32806070 10.1021/acsnano.0c03757

[34] Y. Luo , W. Li , Q. Lin , F. Zhang , K. He , D. Yang , X. J. Loh , X. Chen , Adv. Mater. 2021, 33, 2007848. 10.1002/adma.202007848 33660373

[35] Y. S. Kim , M. Mahmood , Y. Lee , N. K. Kim , S. Kwon , R. Herbert , D. Kim , H. C. Cho , W. H. Yeo , Adv. Sci. 2019, 6, 1900939. 10.1002/advs.201900939 PMC6724359 31508289

[36] a) Y. Xiong , X. Yan , T. Li , H. Jin , Z. Chen , X. Xu , X. Ji , X. Ge , Chem. Eng. J. 2023, 451, 138913;

[37] M. R. Luo , G. Cui , B. Rigg , Color Research & Application 2001, 26, 340.

[38] T. Kari , J. Gadegaard , D. T. Jørgensen , T. Søndergaard , T. G. Pedersen , K. Pedersen , Appl. Opt. 2011, 50, 4860. 21857711 10.1364/AO.50.004860

[39] X.‐Y. Zhang , T. Li , G.‐F. Tan , Y. Huang , F. Wang , A.‐S. Xiong , Plant Growth Regul. 2018, 85, 293.

[40] S. Coste , C. Baraloto , C. Leroy , É. Marcon , A. Renaud , A. D. Richardson , J.‐C. Roggy , H. Schimann , J. Uddling , B. Hérault , Ann. For. Sci. 2010, 67, 607.

[41] M. G. Guerret , E. P. Nyalugwe , S. Maina , M. J. Barbetti , J. A. Van Leur , R. A. Jones , Plant disease 2017, 101, 674. 30678573 10.1094/PDIS-08-16-1129-RE

[42] a) L. Limantara , M. Dettling , R. Indrawati , Indriatmoko, T. H. P. B. , Procedia Chem 2015, 14, 225;

[43] J. D. Barnes , L. Balaguer , E. Manrique , S. Elvira , A. Davison , Environ. Exp. Bot. 1992, 32, 85.

[44] H. L. Gorton , C. R. Brodersen , W. E. Williams , T. C. Vogelmann , Photochem. Photobiol. 2010, 86, 1076. 20553406 10.1111/j.1751-1097.2010.00761.x

[45] F. Cillo , I. M. Roberts , P. Palukaitis , J. Virol. 2002, 76, 10654. 12368307 10.1128/JVI.76.21.10654-10664.2002 PMC136603

