# Supplementary material for: A Leaf‐Patchable Reflectance Meter for In Situ Continuous Monitoring of Chlorophyll Content
Source: Adv Sci (Weinh). 2023 Oct 5;10(35):2305552. doi: 10.1002/advs.202305552 (PMC10724420; doi:10.1002/advs.202305552)
Supplement: Supplementary file 1 — Supporting Information [file ADVS-10-2305552-s002.pdf]

ADVANCED SCIENCE
Open Access

Supporting Information

for *Adv. Sci.*, DOI 10.1002/advs.202305552

A Leaf-Patchable Reflectance Meter for In Situ Continuous Monitoring of Chlorophyll Content

*Kaiyi Zhang, Wenlong Li, Haicheng Li, Yifei Luo, Zheng Li, Xiaoshi Wang and Xiaodong Chen**

Supporting Information

A Leaf-Patchable Reflectance Meter for In Situ Continuous Monitoring of Chlorophyll Content in Plants

*Kaiyi Zhang, Wenlong Li, Haicheng Li, Yifei Luo, Zheng Li, Xiaoshi Wang, Xiaodong Chen**

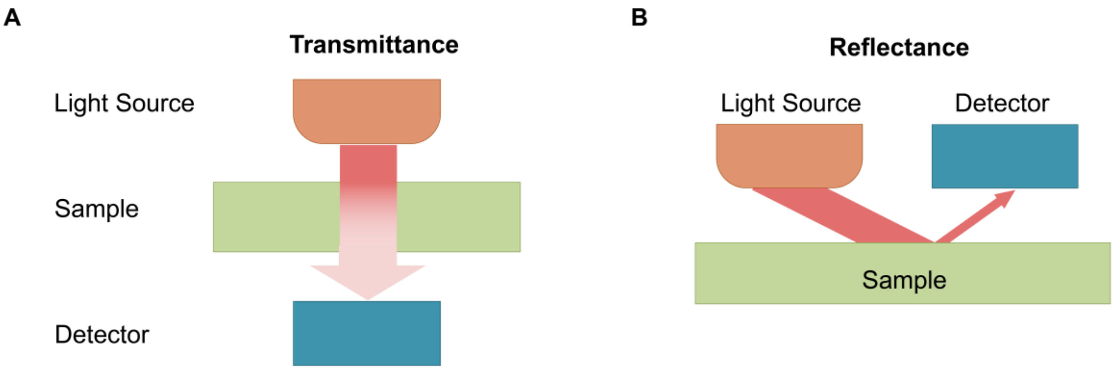

Figure S1. Illustrations of two modes of optical characterization of a leaf sample. A) Transmittance mode. Optoelectronic components have to be placed at different sides of leaves. B) Reflectance mode. Planar layout of all electronic components at one side of leaves can be realized. Reflectance based mechanism was selected for our device due to its planar layout.

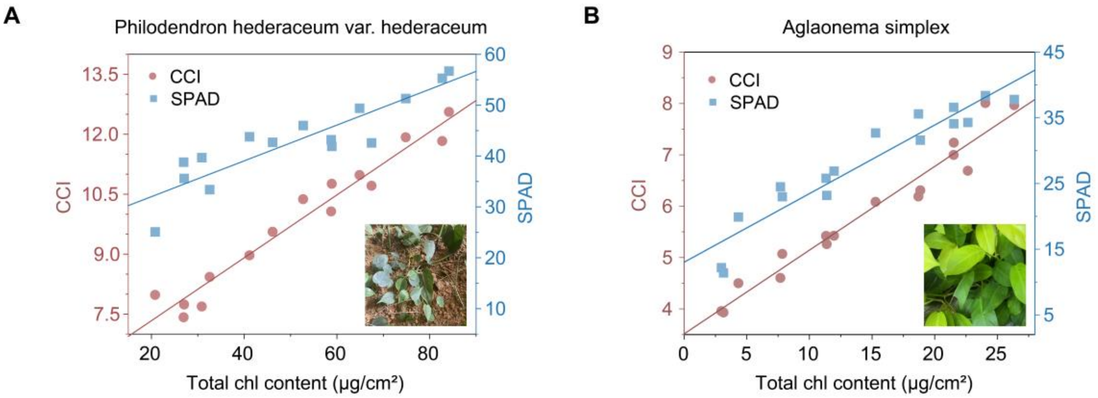

Figure S2. Linear relationships of leaf total chlorophyll concentrations with SPAD value and CCI value on A) Philodendron hederaceum and B) Aglaonema simplex. The CCI calculated with our sensor shows better linearity than SPAD values in commercial products.

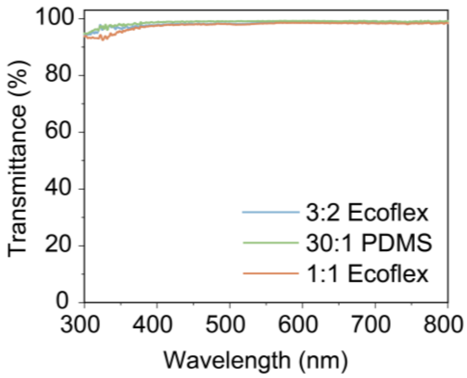

Figure S3. Transmittance spectra of three soft polymeric materials from 300 nm to 800 nm.

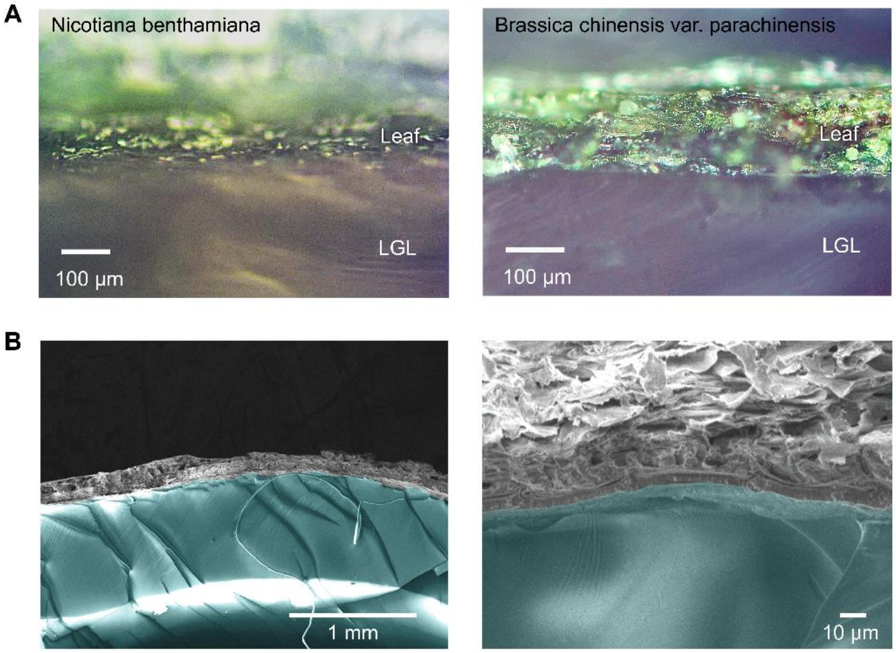

Figure S4. A) Cross-sectional optical microscopy images of LGL attached to the upper epidermis of a *Nicotiana benthamiana* and *Brassica chinensis* var. *parachinensis* leaf. B) Cross-sectional scanning electron microscopy images of LGL attached to the upper epidermis of a bent *Monstera deliciosa* leaf.

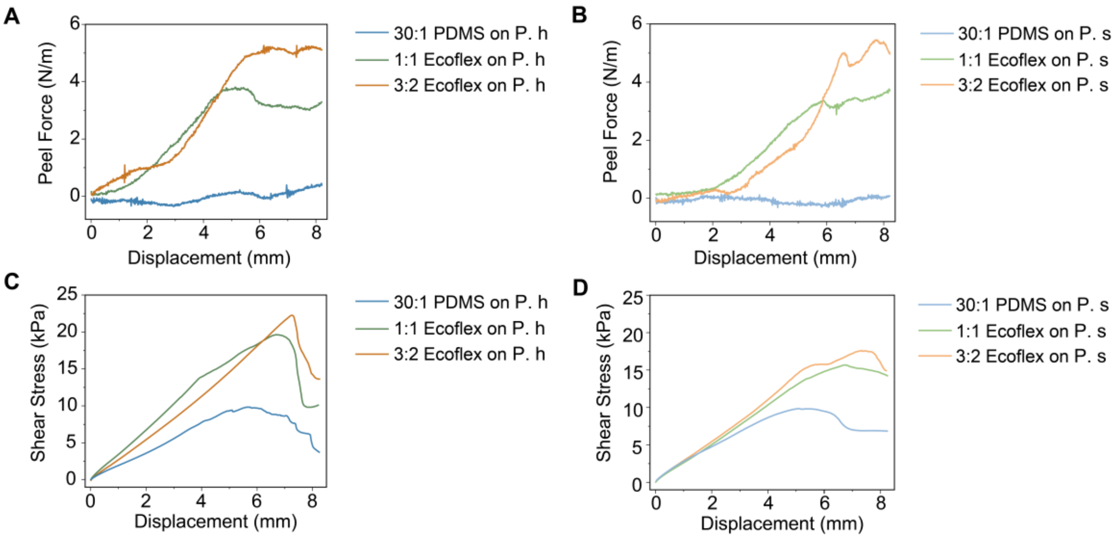

Figure S5. Representative peel force-displacement and shear stress-displacement curves. A) Peel force-displacement of LGL made of different soft polymeric materials on leaves of *Philodendron hederaceum*. B) Peel force-displacement of LGL made of different soft polymeric materials on leaves of *Piper sarmentosum*. C) Shear stress-displacement of LGL made of different soft polymeric materials on leaves of *Philodendron hederaceum*. D) Shear stress-displacement of LGL made of different soft polymeric materials on leaves of *Piper sarmentosum*.

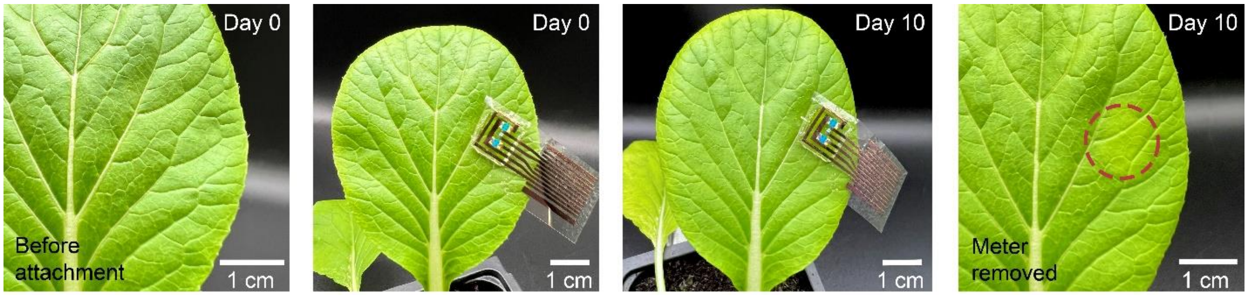

Figure S6. Leaves of *Brassica chinensis* var. *parachinensis* before our meter is attached, after our meter is attached for 10 days, and after our meter is removed.

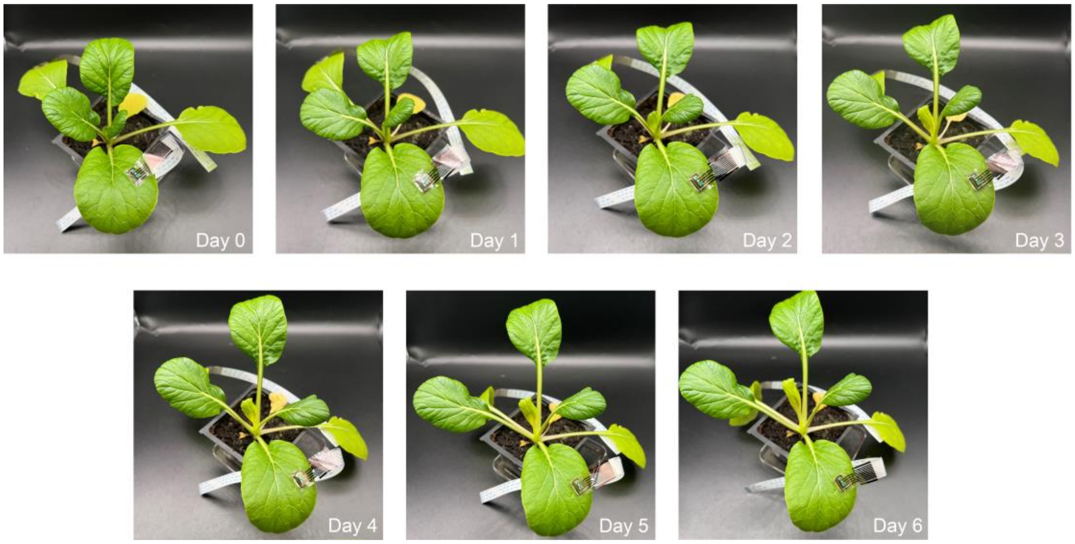

Figure S7. Photographs of *Brassica chinensis* var. *parachinensis* under dark stress from day 0 to day 6. Color difference was calculated based on these photos. Photos were taken with the same photographic parameters (ISO 80, 26 mm, f1.5, 1/100 s).

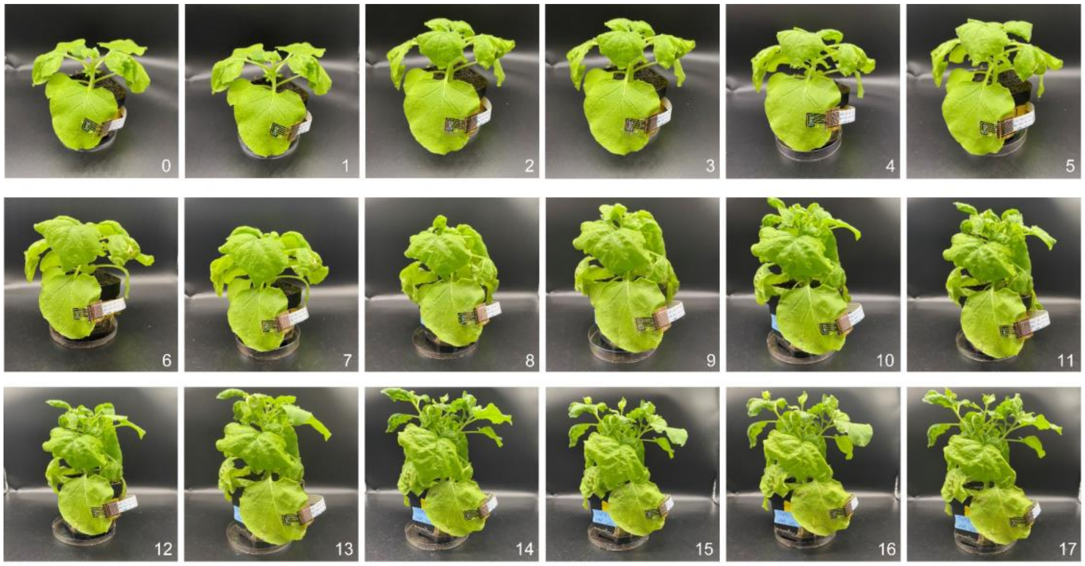

Figure S8. Photographs of *Nicotiana benthamiana* infected by CMV from 0 dpi to 17 dpi. Photos were taken with same photographic parameters (ISO 100, 26 mm, f1.8, 1/50 s). Color difference was calculated based on these photos.

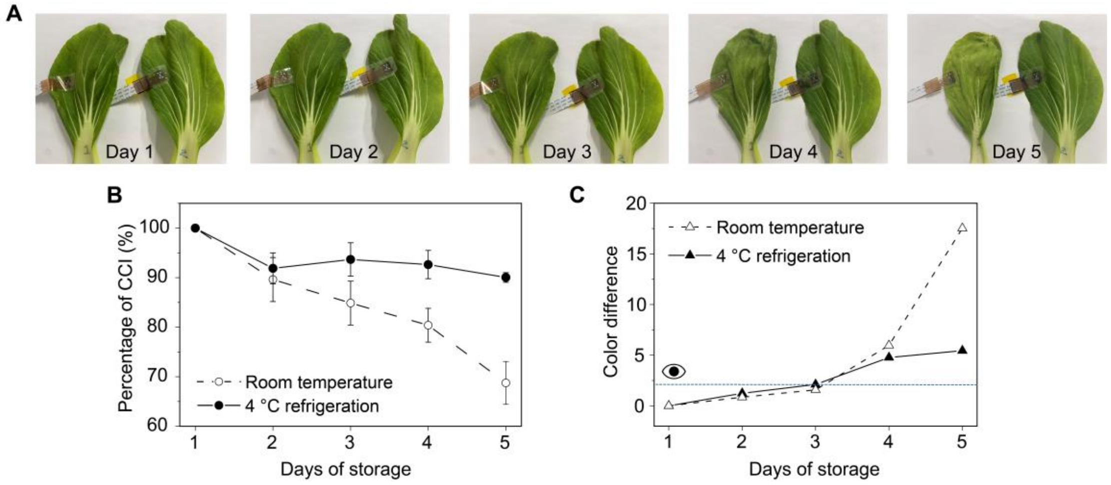

Figure S9. Potential application of the wearable chlorophyll sensor in detecting the freshness of green leafy vegetables. A) Photographs of bok choy stored in room temperature (left) and 4 ℃ refrigerator (right) for 5 days. B) Percentage of CCI value of both groups in 5 days. C) Color difference of both groups in 5 days. The dark blue dotted line with an eye above refers to JND. In first three days, the color differences of both groups were lower than JND, and obvious color difference between two groups could only be observed at day 5. Our sensor can quantitatively detect the obvious decrease of freshness of bok choy at room temperature in day 3.

Table S1.

Comparison between commercial SPAD meter and our sensor

	SPAD meter	Our Sensor
Function	Chlorophyll measurement	Chlorophyll measurement
Principle	Leaf transmittance	Leaf reflectance
Form	Rigid, handheld	Morphable, wearable
Size	78 (W) × 164 (H) × 49 (D) mm	20 (W) × 40 (H) × 1.5 (D) mm
Weight	200 g	0.2 g
Application	One-time, in-filed measurement	Long-term, in situ monitoring

Movie S1.

Measuring CCI under different light environment.
Ambient light: 6.72, 6.71, 6.71;
Dark: 6.69, 6.69, 6.69;
Strong light: 6.69, 6.68, 6.71

The wireless platform for CCI measuring can be downloaded via the link below:
https://github.com/ZeKaylee/CCI/tree/main
